# Suicidality and its associated factors among mood disorder patients in emergency department in China: a comparative study using propensity score matching approach

**DOI:** 10.1038/s41398-023-02675-0

**Published:** 2023-12-01

**Authors:** Xiao-meng Xie, Yi-Fan Wang, Tian Han, Yi Liu, Juan Li, Hui Zhu, Tao Jiang, Xiao Ji, Hong Cai

**Affiliations:** 1 Beijing Key Laboratory of Mental Disorders, National Clinical Research Center for Mental Disorders & National Center for Mental Disorders, Beijing Anding Hospital, & the Advanced Innovation Center for Human Brain Protection, Capital Medical University, Beijing, China; 2 Unit of Medical Psychology and Behavior Medicine, School of public health, Guangxi Medical University, Nanning, Guangxi China

**Keywords:** Bipolar disorder, Depression

## Abstract

Suicidality in mood disorder patients is common, especially in emergency department (ED), but the patterns and associated factors of suicidality are not clear. This study compared biomarkers and mental health symptoms (i.e., depression, anxiety, and psychiatric symptoms) between mood disorder patients with and without the whole range of suicidality comprising suicidal ideation (SI), suicide plan (SP), and suicide attempt (SA). This cross-sectional, comparative, convenient-sampling study was conducted between January 2021 and March 2022, in emergency department of Beijing Anding Hospital, China. Patients with mood disorders at a psychiatric emergency department were assessed, with measurements of suicidality, biomarkers, depressive, anxiety, and psychiatric symptoms were assessed using the 24 items-Hamilton Depression Rating Scale (HAMD-24), Hamilton Anxiety Rating Scale (HAMA), Young Manic Rating Scale (YMRS) and Brief Psychiatric Rating Scale (BPRS), respectively. The propensity score matching (PSM) method was used to identify patients in mood disorder with and without SI, SP, and SA. A generalized linear model (GLM) was used to assess the differences in biomarkers, depressive, anxiety, and psychiatric symptoms between patients in mood disorder with and without SI, SP, and SA. In total, 898 participated in this survey and completed the assessment. Illness duration was significantly negatively associated with SA (OR = 0.969, 95%CI = 0.939–0.999, *P* = 0.046). HAMD-24 total score was significantly positively associated with the SI (OR = 1.167, 95%CI = 1.134–1.201, *p* < 0.001), SP (OR = 1.159, 95%CI = 1.126–1.192, *p* < 0.001) and SA (OR = 1.189, 95%CI = 1.144–1.235, *p* < 0.001) of the matched samptched sample. However, YMRS total score was significantly negatively associated with the SI (OR = 0.928, 95%CI = 0.905–0.951, *p* < 0.001), SP (OR = 0.920, 95%CI = 0.897–0.944, *p* < 0.001) and SA (OR = 0.914, 95%CI = 0.890–0.938, *p* < 0.001) of the matched sample after adjusting for age, gender, marital status, and occupation. The duration of illness, severity of depressive symptoms and severity of manic symptoms appeared to be more likely to influence suicidality. Considering the significant risk of suicide in mood disorders on psychiatric emergency care, timely treatment and effective management of suicidality in this population group need to be developed.

## Introduction

Suicide, responsible for an estimated 703,000 deaths worldwide each year, remains a significant public health issue [1]. Beyond the immediate personal and familial distress that it causes, suicide also amplifies the healthcare burden. While various factors can lead to suicide in the general population, the presence of psychiatric conditions, especially mood disorders, elevates the risk significantly. As a lifelong episodic illness, mood disorder can result in various setbacks, from functional, occupational, and cognitive impairments [2] to severe outcomes like suicidality [3]. Notably, emergency department (ED) visits in the USA related to suicidality more than doubled from 244,000 in 1993–1996 to 538,000 in 2005–2008 [4]. Furthermore, an estimated one in ten patients exhibiting suicidality had visited an emergency room within two months preceding their death [5]. Compared to those without psychiatric disorders, individuals with mood disorders exhibit substantially higher rates of suicidality [6], sparking widespread international concern.

Suicidal behaviors, which collectively escalate the risk for suicide, include suicidal ideation (SI), suicide plans (SP), and suicide attempts (SA) [3, 7]. SI, colloquially termed as suicidal thoughts, encompasses all suicidal considerations without necessarily progressing to more overt acts. SP pertains to the strategizing of self-harm with the intent to end one’s life [8], while SA involves actual actions taken with this intent. Within the context of major depressive depression (MDD), the prevalence of SI is observed to be 37.7% (95% CI: 32.3–43.4%), whereas SP is reported at a pooled prevalence of 15.1% (95% CI: 8.0–26.8%) [9]. SI, SP, and SA are often predictors of eventual suicide, forming a spectrum characterized by escalating psychological distress and neuroendocrine alterations [10]. Notably, around one-third of adolescents who exhibit SI eventually develop SP, and close to 60% with SP transition to SA [11]. Given the diverse prevalence rates, clinical manifestations, and outcomes of these behaviors, there remains an uncharted territory regarding their patterns and associated factors within ED settings. As the ED serves as a pivotal interface in the healthcare system, the immediate need is to devise proficient screening and risk assessment mechanisms integrating multiple biomarkers, thereby ensuring efficacious suicide prevention during ED consultations [12].

A substantial body of evidence implicates inflammation, nutrition, and neurohormones as significant contributors to the genesis of suicidality (Oliveira et al., 2017). Specifically, alterations in immune markers like C-reactive protein (CRP) have been identified in the peripheral blood. Notably, MDD patients consistently show elevated CRP levels compared to their non-affected counterparts [13–15]. The hormones cortisol and adrenocorticotropic hormone (ACTH) offer critical insights into the functioning of the hypothalamic-pituitary-adrenal (HPA) axis. Disrupted secretion patterns of these hormones are discernible across MDD [16], BD [17], and Schizophrenia (SCZ) [18] which are further consolidated by meta-analyses [17, 19]. The most consistent observation relating to suicide has been its association with HPA axis anomalies. Moreover, recent research suggests testosterone, a sex hormone, plays a partial role in modulating suicidal behaviors [20]. In light of this, we have incorporated these elements as prospective biomarkers in our investigation.

Traditionally, suicidality in mood disorders is conceptualized as observable expressions of a single underlying condition. This perspective, however, tends to oversimplify by overlooking the unique causality, progression, and heterogeneity of individual symptoms. In traditional methodology approach, suicidality was acted as the dependent variable and effected by many covariates. It also neglects the intricate interrelations among these symptoms [21]. Enter the Propensity Score Matching (PSM) method, an innovative approach for balancing covariates between experimental and control groups, especially when an ideal randomized experimental design is unfeasible [22]. PSM stands out for its capability to discern the influence of an independent variable while substantially reducing the selection bias, thereby ensuring the validity of the results [22–24]. Recognizing its advantages, we incorporated PSM in our study to equate demographic variances between mood disorder patients, segmented based on the presence or absence of SI, SP, and SA. Such methodological refinement elevates our capacity to estimate suicidality risk in vulnerable individuals. The aim of this study was to provide the potential targets for warranting the physicians’ attention in the evaluation protocol.

## Methods

### Study setting and participants

This cross-sectional comparative study was conducted between January 2021 and March 2022, in emergency department of Beijing Anding Hospital, which has a 300-bed Mood Disorders Centre. The Psychiatric Emergency Department is divided into an emergency treatment area consisting of a 17-bed emergency observation ward and 4 ICU beds. The average number of emergency room visits per month was 2000. It provides the only 24-h emergency service for psychiatric hospitals in Beijing and neighboring provinces in North China.

Patients attending the emergency outpatient clinics were invited to participate in the study. To be eligible, participants needed to meet the following criteria: 1) aged 18 years or older; 2) diagnosed with mood disorder according to the F30-39 section of the tenth revision of the International Classification of Disease (ICD-10) [25] by a consensus of two senior psychiatrists; 3) able to understand the aim and contents of the assessment and provide verbal informed consent. Persons with cognitive impairment were excluded. The study was conducted on a voluntary and confidential basis and the study protocol was approved by the Ethics Committee of Beijing Anding Hospital.

### Data collection and assessment tools

A data collection form was used to collect demographic information, including age, gender, education level, occupation, living status, marital status, medical insurance, family history of mental health, health status, relationship with friends or family members, whether irritability is a personal characteristic, family support, whether a stress event occurred, and interpersonal communication. Clinical data included illness duration (years) and age at first episode. The 24-item Hamilton Depression Rating Scale (HAMD-24) was used to assess depressive symptoms. The Chinese version of the HAMD-24 has been validated with satisfactory psychometric properties (e.g., Cronbach’s alpha of *α* = 0.92) [26]. The Hamilton Anxiety Rating Scale (HAMA) was used to assess anxiety symptoms. The Chinese version of the HAMA has been validated with satisfactory psychometric properties (e.g., Cronbach’s alpha of *α* = 0.93) [27]. The Young Mania Rating Scale (YMRS) is one of the most frequently utilized rating scales to assess manic symptoms [28]. The Brief Psychiatric Rating Scale (BPRS) [29] was used to estimate psychiatric symptoms. The Chinese version of the BPRS has been validated with satisfactory psychometric properties [30]. These psychiatrists were trained on the assessment of HAMD, HAMA, YMRS, and BPRS in a clinical study before the study began. The inter-rater correlation coefficient of HAMD, HAMA, BPRS, and PANSS scores of psychiatrists were all more than 0.8.

In this study, current SI, SP, and SA were assessed through face-to-face interview. SI was assessed with a standard question (“Have you thought that you would be better off dead currently?”) that included a binary response option (yes/no). SP was assessed with a standard question (“Have you made a plan for suicide currently?”) featuring a binary response option (yes/no). SA were evaluated with a standard question (“Have you attempted suicide currently?”), including a binary response option (yes/no).

### Biochemical parameters measurements

All subjects had their peripheral blood taken before 10 o’clock in the morning within 24–72 h after admission to the hospital, and some participants also received their blood test before being discharged from the hospital. Fasting biochemical indexes were measured, including CRP, cortisone, ACTH, and testosterone were collected from medical records available in the hospital.

### Statistical analyses

#### Propensity score matching

Due to different demographic characteristics between the mood disorder patients with suicidality and non-suicidality patients in this study, the optimal fixed ratio matching based on propensity scores was used to identify comparable suicidality and non-suicidality mood disorders patients with a matching ratio of 1:1. Propensity score is the probability of a participant being assigned to a particular group (i.e., age, gender, marital status and occupation in this study), calculated by a logistic regression model based on a given set of observed covariates (i.e., confounders) [31]. The propensity score matching procedure would match each participant in suicidality groups with one non-suicidality, thereby balancing the potential confounders between the two groups [31, 32]. The propensity score analysis could help reduce bias in research results by minimizing the confounding effects caused by unmatched demographic characteristics [32]. Confounders refer to variables that affect both the outcome variable and the grouping variable; the potential confounders matched in the propensity score model are selected based on the variable-grouping relationships and the variable-outcome relationships [32, 33]. In this study, the variable-grouping relationships and the variable-outcome relationships were assessed using independent two-sample *t* tests, Wilcoxon rank-sum tests, and chi-square tests as appropriate. Confounders were selected based on an expert consensus and the findings of previous studies in the propensity score model [34].

### Univariable analyses

In univariable analyses before and after matching, the demographic and clinical characteristics between the MD patients with suicidality to those without suicidality. Suicidality MD patients and non-suicidality MD patients were compared using independent two-sample *t* tests, Wilcoxon rank-sum tests, and *χ*^2^ tests as appropriate. In the matched study sample, demographic characteristics that were significant in the univariable analyses were adjusted for in multivariable analysis models.

### Multivariable analyses

In the matched study sample, the binary logistic regression was used to examine independent correlates of suicide behaviors. SI, SP, and SA were entered as separate dependent variables while measures with significant suicidality versus no suicidality group differences in univariate analyses were independent variables, while adjusting for the demographic characteristics that were still significant in the univariable analyses after matching. All data analyses were conducted using the “MatchIt” function of R [35]. Two-tailed *p* values <0.05 were considered as statistically significant.

## Results

### Demographic and clinical characteristics of the whole sample

Altogether, 898 participants in this survey and completed the assessment. Suicidality and non-suicidality participants were statistically different in gender occupation, unmarried, irritability personal characteristics, stress event, less interpersonal communication, age, education background, illness duration, first episode age, Cortisone, ACTH, sex, Testosterone, HAMD-24 total, HAMA total, YMRS total and BRPS total (all *p* values < 0.05; Supplementary Table 1).

### Potential confounder selection for propensity score matching

According to the preliminary results of the variable-grouping and the variable-outcome relationships (Supplementary Tables 1), occupation, and marital status of the MD patients were selected as the potential confounders, all of which were matched in the propensity score model. Additionally, since age and gender were the most commonly used confounders in previous studies [36–38], age and gender were also selected for matching in the propensity score model.

The propensity scores matching procedure identified 280 comparable patients with SI and patients without SI in each group, composing a matched study sample of 560 participants. The propensity scores matching procedure identified 273 comparable patients with suicide plan and patients without suicide plan in each group, composing a matched study sample of 546 participants. The propensity scores matching procedure identified 275 comparable patients with suicide attempt and patients without suicide attempt in each group, composing a matched study sample of 550 participants.

### Univariate and multivariate analysis of the matched sample

The demographic and clinical characteristics of the matched two samples are shown in Table 1. Univariate analyses revealed that the patients with SI and their matched patients without SI were comparable in age, sex, occupation, and marital status (all *p* values > 0.05). There were significant differences between the patients with SI and patients without SI in terms of the irritability personal characteristics, stress event, less interpersonal communication, education background, illness duration, HAMD-24 total, HAMA total, and YMRS total scores (all *p* values < 0.05; Supplementary Figs. 1–3).

**Table 1:** Demographic and clinical characteristics of the matched sample.

Variables	Suicidal ideation					Suicide plan					Suicide attempt				
	No(*N* = 280)	Yes(*N* = 280)	Statistics			No(*N* = 273)	Yes(*N* = 273)	Statistics			No(*N* = 275)	Yes(*N* = 275)	Statistics		
	*N* (%)	*N* (%)	*X*^2^	df	*p* value	*N* (%)	*N* (%)	*X*^2^	df	*p* value	*N* (%)	*N* (%)	*X*^2^	df	*p* value
Male gender	95 (33.9)	95 (33.9)	0.00	1	1.00	91 (33.3)	90 (33.0)	0.01	1	0.93	97 (35.0)	92 (33.5)	0.26	1	0.61
Occupation	255 (91.1)	257 (91.8)	0.09	1	0.76	253 (92.7)	253 (92.7)	0.00	1	1.00	254 (91.7)	254 (92.4)	0.18	1	0.67
Urban	224 (80.0)	216 (77.1)	0.68	1	0.41	218 (79.9)	215 (78.8)	0.10	1	0.75	221 (79.8)	215 (78.8)	0.09	1	0.77
Medical insurance	200 (71.4)	192 (68.6)	0.54	1	0.46	197 (72.2)	189 (69.2)	0.57	1	0.45	201 (72.6)	191 (69.5)	0.74	1	0.39
Unmarried	152 (54.3)	150 (53.6)	0.03	1	0.87	152 (55.7)	151 (55.3)	0.01	1	0.93	155 (56.0)	151 (54.9)	0.02	1	0.88
Family history	72 (25.7)	69 (24.6)	0.09	1	0.77	67 (24.5)	66 (24.2)	0.01	1	0.92	67 (24.2)	67 (24.2)	0.00	1	1.00
Good health status	221 (78.9)	214 (76.4)	0.51	1	0.78	212 (77.7)	210 (76.9)	0.10	1	0.95	215 (77.6)	211 (76.7)	0.11	1	0.95
Poor relationship with friends/family members	118 (42.1)	130 (46.4)	1.04	1	0.31	114 (41.8)	125 (45.8)	0.90	1	0.34	118 (42.6)	126 (45.8)	0.57	1	0.45
Irritability personal characteristics	174 (62.1)	223 (79.6)	20.78	1	**<0.001**	167 (61.2)	216 (79.1)	21.00	1	**<0.001**	170 (61.4)	218 (79.3)	20.70	1	**<0.001**
Poor family support	68 (24.3)	61 (21.8)	0.49	1	0.48	66 (24.2)	57 (20.9)	0.85	1	0.36	69 (24.9)	58 (21.1)	1.27	1	0.26
Stress event	33 (11.8)	67 (23.9)	14.07	1	**<0.001**	34 (12.5)	63 (23.1)	10.54	1	**0.001**	33 (11.9)	63 (22.9)	11.89	1	**0.001**
Less interpersonal communication	170 (60.7)	217 (77.5)	18.48	1	**<0.001**	166 (60.8)	213 (78.0)	19.06	1	**<0.001**	169 (61.0)	214 (77.8)	18.75	1	**<0.001**

	Mean (SD)	Mean (SD)	t/z	df	*p* value	Mean (SD)	Mean (SD)	t/z	df	*p* value	Mean (SD)	Mean (SD)	t/z	df	*p* value
Age (years)	35.35 (17.44)	34.44 (18.83)	−1.44	–^a^	0.15	35.70 (17.60)	34.00 (18.87)	−2.05	–^a^	**0.04**	35.82 (17.58)	34.12 (18.91)	−2.03	–^a^	**0.04**
Education background (years)	13.18 (3.57)	12.50 (3.79)	2.18	558	**0.03**	13.21 (3.65)	12.42 (3.75)	2.49	544	**0.01**	13.19 (3.64)	12.45 (3.76)	2.34	548	**0.02**
Illness duration (years)	7.34 (8.68)	5.73 (7.60)	−2.47	–^a^	**0.01**	7.48 (8.90)	5.83 (7.66)	−2.03	–^a^	**0.02**	7.52 (8.97)	5.79 (7.65)	−2.37	–^a^	**0.02**
First episode age (years)	28.23 (15.15)	28.69 (17.19)	−0.99	–^a^	0.32	28.56 (15.17)	28.15 (17.09)	−1.76	–^a^	0.08	28.63 (15.16)	28.31 (17.19)	−1.70	–^a^	0.09
CRP (μg/L)	0.57 (1.88)	0.59 (1.34)	−0.37	–^a^	0.71	0.58 (1.92)	0.59 (1.36)	−0.06	–^a^	0.95	0.59 (1.93)	0.59 (1.35)	−0.20	–^a^	0.84
Cortisone (nmol/L)	16.06 (8.78)	15.61 (9.14)	−0.58	–^a^	0.56	16.38 (8.78)	15.29 (9.27)	−1.48	–^a^	0.14	16.25 (8.70)	15.31 (9.25)	−1.28	–^a^	0.20
ACTH (pg/ml)	32.11 (30.19)	27.81 (25.03)	−1.17	–^a^	0.24	32.81 (30.12)	27.49 (25.14)	−1.78	–^a^	0.08	32.48 (29.82)	27.61 (25.17)	−1.66	–^a^	0.10
Testosterone (nmol/L)	166.20 (210.96)	146.53 (195.50)	−1.14	–^a^	0.25	166.40 (212.83)	142.91 (193.83)	−1.37	–^a^	0.17	170.84 (216.68)	143.65 (193.39)	−1.46	–^a^	0.14
HAMD-24 total	9.06 (7.92)	23.32 (10.03)	−18.67	–^a^	**<0.001**	9.21 (8.29)	23.10 (9.98)	−14.86	–^a^	**<0.001**	9.31 (8.33)	23.05 (9.96)	−14.80	–^a^	**<0.001**
HAMA total	5.13 (6.92)	12.82 (8.59)	−12.12	–^a^	**<0.001**	5.42 (6.87)	12.55 (8.44)	−11.36	–^a^	**<0.001**	5.42 (6.82)	12.54 (8.41)	−11.43	–^a^	**<0.001**
BPRS total	32.13 (11.30)	31.69 (9.37)	0.51	558	0.61	32.26 (11.45)	31.40 (9.31)	0.97	544	0.33	32.34 (11.25)	31.40 (9.28)	1.07	548	0.29
YMRS	16.67 (11.78)	6.96 (7.52)	−9.75	–^a^	**<0.001**	16.51 (11.73)	6.67 (7.31)	−9.93	–^a^	**<0.001**	16.75 (11.64)	6.68 (7.31)	−10.27	–^a^	**<0.001**

Bolded value: <0.05.

*CRP* C-reactive protein, *ACTH* adreno-cortico-tropic-hormone, *HAMD* Hamilton Depression Rating Scale, *HAMA* Hamilton Anxiety Rating Scale, *YMRS* Young Manic Rating Scale, *BPRS* The Brief Psychiatric Rating Scale.

Univariate analyses revealed that the patients with suicide plan and their matched patients without SP were comparable in age, sex, occupation, and marital status (all *p* values > 0.05). There were significant differences between the patients with SP and patients without SP in terms of the irritability personal characteristics, stress event, less interpersonal communication, lower education background, illness duration, HAMD-24 total, HAMA total, and YMRS total scores (all *p* values < 0.05).

Univariate analyses revealed that the patients with SA and their matched patients without SA were comparable in age, sex, occupation, and marital status (all *p* values > 0.05). There were significant differences between the patients with SA and patients without SA in terms of the irritability personal characteristics, stress event, less interpersonal communication, education background, illness duration, HAMD-24 total, HAMA total, and YMRS total scores (all *p* values < 0.05).

Table 2 presents results of binary logistic regression analyses. There are significant correlation (*p* < 0.001) between HAMD-24 total score and HAMA total score among the SI (*p* < 0.001, OR: 1.167, 95%CI: 1.134–1.201), SP (*p* < 0.001, OR: 1.159, 95%CI: 1.126–1.192) and SA (*p* < 0.001, OR: 1.189, 95%CI: 1.144–1.235) of the matched sample, respectively. Illness duration was significantly negatively associated with SA (OR = 0.969, 95%CI = 0.939–0.999, *p* = 0.046). HAMD-24 total score was significantly positively associated with the SI (OR = 1.167, 95%CI = 1.134–1.201, *p* < 0.001), SP (OR = 1.159, 95%CI = 1.126–1.192, *p* < 0.001) and SA (OR = 1.189, 95%CI = 1.144–1.235, *p* < 0.001) of the matched sample. However, YMRS total score was significantly negatively associated with the SI (OR = 0.928, 95%CI = 0.905–0.951, *p* < 0.001), SP (OR = 0.920, 95%CI = 0.897–0.944, *p* < 0.001) and SA (OR = 0.914, 95%CI = 0.890–0.938, *p* < 0.001) of the matched sample (Table 2).

**Table 2:** Independent correlates of suicidality by multiple logistic regression analyses.

Variables	Suicidal ideation			Suicide plan			Suicide attempt		
	*p* value	OR	95%CI	*p* value	OR	95%CI	*p* value	OR	95%CI
Irritability personal characteristics	0.39	1.29	0.72–2.32	0.36	1.31	0.73–2.35	0.27	1.39	0.77–2.48
Stress event	0.78	1.09	0.59–2.03	0.93	0.97	0.52–1.81	0.71	1.13	0.60–2.14
Less interpersonal communication	0.52	1.20	0.68–2.13	0.49	1.23	0.69–2.19	0.60	1.17	0.65–2.10
Education background (years)	0.51	0.98	0.92–1.04	0.44	0.98	0.92–1.04	0.57	0.98	0.92–1.04
Illness duration (years)	0.09	0.97	0.94–1.00	0.08	0.97	0.94–1.00	**0.05**	**0.97**	**0.94–1.00**
HAMD-24 total	**<0.001**	**1.17**	**1.13–120**	**<0.001**	**1.16**	**1.13–1.19**	**<0.001**	**1.19**	**1.14–1.24**
YMRS total	**<0.001**	**0.93**	**0.91–0.95**	**<0.001**	**0.92**	**0.90–0.94**	**<0.001**	**0.91**	**0.89–0.94**

Bolded value: <0.05.

*HAMD* Hamilton Depression Rating Scale, *YMRS* Young Manic Rating Scale, *CI* confidential interval, *OR* odds ratio.

## Discussion

In this study conducted in China’s Emergency departments, we observed for the first time that patients with mood disorders and suicidality presented with higher HAMD total scores, increased symptoms of hypomania, and a shorter duration of illness compared to their non-suicidal counterparts with mood disorders. Notably, there were no significant disparities in anxiety levels, inflammation markers like CRP, or biological indicators such as Cortisol and ACTH levels.

Early in the course of the disease, the risk of the suicidality is peak. Consistently, our findings depicted a negative correlation between the duration of the illness and SA. This contrasts with previous research that suggested an association between longer illness duration and more severe outcomes in BD, especially in the context of suicidality [39]. Upon being diagnosed with a mood disorder, patients may grapple with feelings of hopelessness, an overwhelming symptom burden, diminished overall functionality, and an increased reliance on mental health services. Any of these factors could precipitate suicidal attempts.

Moreover, while sociocultural elements play a part, previous studies indicate that the increased risk of suicidality in patients with a short illness duration might be linked to biological factors. These include disrupted N-acetylaspartate (NAA)-glutamatergic metabolism in the anterior cingulate cortex [40], and compromised executive functioning and impulse control due to decreased structural connectivity in the frontal subcortical circuit [41].

Among mood disorder inpatients, the severity of depressive symptoms was significantly positively associated with SI, SP, and SA, which is also true of MDD patients [9, 42, 43]. This pattern can probably be attributed to the more severe psychotic symptoms [44, 45] and somatic symptoms [46] among mood disorders patients. Recent findings also indicate that more severe psychiatric symptoms are linked to higher suicide risk in both the MDD and general population and for those with pre-existing psychiatric conditions during the pandemic [47, 48]. Residual psychiatric symptoms such as depressive symptoms may be reflections of past suicidality or serve as prognosticators for worse outcomes, including increased risk for relapse, recurrence, and suicidality [49, 50]. Hence, these symptoms should be addressed in maintenance treatment and rehabilitation for patients when they visit emergency department.

One reason might be that inpatients may have hallucinations commanding them to self-harm. Moreover, mood disorders patients tend to have more severe and multimorbid psychopathology, a higher burden of symptoms, poorer global functioning, and higher usage of mental health services, all of which were associated with SI, SP, and SA. The severity of depressive symptoms is also a manifestations of illness episodes which has recently been shown to be associated with occurrence of suicide attempts [51]. As expected, patients with depressive course of illness have a 2-fold risk of suicide attempts, compared with predominantly manic patients; including patients with mixed states into depressive polarity strengthens the association to 4.5-fold [51]. Recent findings also indicate that more severe psychiatric symptoms are linked to higher suicide risk in both the general population and those with pre-existing psychiatric conditions during the pandemic [52]. Residual psychiatric symptoms such as fatigue, pain, and depressive symptoms may be reflections of past suicidality or serve as prognosticators for worse outcomes, including increased risk for relapse, recurrence, and suicidality [49, 50]. Hence, these symptoms should be addressed in maintenance treatment and rehabilitation for clinically stable psychiatric patients during the pandemic.

In our study, we found that the severity of manic symptoms was negatively related to the SI, SP, and SA among mood disorders patients. The result was consistent with the previous findings that hypomania symptoms have been related to mood instability, impulsive behaviors, and increased risk of suicidality in individuals with bipolar disorder [53–55]. In the same line, suicidality was related to manic, depressive symptoms and emotion dysregulation, especially to emotional impulsivity and to difficulties in regulatory strategies, as previously observed [56]. Indeed, emotional impulsivity and manic symptoms were the factors more strongly related to suicidality. Manic symptoms as a component of one type of emotion dysregulation is defined as an impairment in the modulation of some aspects of emotional functioning including early emotional processes, the appraisal and evaluation of stimuli and emotional response with its behavioral and physiological components in both the immediate context and in the long-term objectives/goals of individuals [55]. Furthermore, our included participants were patients who seek medical help in an emergency center and were in the episode state.

The strengths of this study included using the propensity score matching method and collecting participants in emergency department. However, several limitations should be noted. First, patients were the inpatients who presented to the emergency department, psycho-social variables might be more serious than other department patients, which might have recall and reporting bias. Second, the data were collected based on self-report; therefore, the possibility of recall bias could not be excluded. Third, for logical reasons, this was cross-sectional research that was unable to assess the causal relationships among study variables. Longitudinal studies closely examining the development of psychiatric disorders, illness episode, cooccurring disorders, and suicide, are needed to answer these crucial questions of causation. Fourth, the analyses primarily made use of dichotomous dependent variables (e.g., have or have not attempted suicide), providing no information regarding the severity of assigned suicidal ideation or attempts. Finally, the information about the use of psychotropic medications was not recorded.

In conclusion, as most psychiatric patients suffering from a major depressive or a mixed mood episode actually consider, plan, or attempt suicide, mere suffering from a major mood disorder itself explains much of the associated suicidal behaviors. As a promising setting for suicide prevention, the emergency department needs to establish some appropriate strategies for universal screening, risk assessment and follow-up care. However, the temporal and dose-exposure association with severity of illness, duration of illness risk states are the prevention components that need to be focused on.

### Supplementary information

supplementary naterials

## Data Availability

The data of the investigation will be made publicly available if necessary.
