# Supplementary material for: Suicidality and its associated factors among mood disorder patients in emergency department in China: a comparative study using propensity score matching approach
Source: Transl Psychiatry. 2023 Dec 1;13:372. doi: 10.1038/s41398-023-02675-0 (PMC10692218; doi:10.1038/s41398-023-02675-0)

**Supplementary materials**

Figure 1. Distribution of propensity score of suicidal ideations

Figure 2. Distribution of propensity score of suicide plan

Figure 3. Distribution of propensity score of suicide attempts

Supplementary Table 1. Demographic and clinical characteristics of whole sample

| Variables |  | Suicidal ideation | | | | | Suicide plan | | | | | Suicide attempt | | | | |
| --- | --- | --- | --- | --- | --- | --- | --- | --- | --- | --- | --- | --- | --- | --- | --- | --- |
|  | Total  (N=898) | No (N=618) | Yes (N=280) | Statistics | | | No (N=625) | Yes  (N=273) | Statistics | | | No (N=623) | Yes  (N=275) | Statistics | | |
|  | N  (%) | N  (%) | N  (%) | X^2^ | df | p | N  (%) | N  (%) | X^2^ | df | p | N  (%) | N  (%) | X^2^ | df | p |
| Male gender | 366  (40.8) | 271  (43.9) | 95  (33.9) | 7.857 | 1 | **0.005** | 276  (44.2) | 90  (33.0) | 9.859 | 1 | **0.002** | 274  (44.0) | 92  (33.5) | 8.755 | 1 | **0.003** |
| Occupation | 782  (87.1) | 525  (85.0) | 257 (91.1) | 8.001 | 1 | **0.005** | 529  (84.6) | 253  (92.7) | 10.902 | 1 | **0.001** | 528  (84.8) | 254  (92.4) | 9.828 | 1 | **0.002** |
| Urban | 690  (76.8) | 474  (76.7) | 216 (77.1) | 0.021 | 1 | 0.884 | 475  (76.0) | 215  (78.8) | 0.810 | 1 | 0.368 | 475  (76.2) | 215  (78.8) | 0.403 | 1 | 0.526 |
| Medical insurance | 647  (72.0) | 455  (73.6) | 192 (68.6) | 2.443 | 1 | 0.118 | 458  (73.3) | 189  (69.2) | 1.547 | 1 | 0.214 | 456  (73.2) | 191  (69.5) | 1.325 | 1 | 0.250 |
| Unmarried | 398  (44.3) | 248  (40.1) | 150 (53.6) | 14.109 | 1 | **<0.001** | 247  (39.5) | 151  (55.3) | 19.200 | 1 | **<0.001** | 247  (39.6) | 151  (54.9) | 18.009 | 1 | **<0.001** |
| Family history | 238  (26.5) | 169  (27.3) | 69  (24.6) | 0.723 | 1 | 0.395 | 172  (27.5) | 66  (24.2) | 1.091 | 1 | 0.296 | 171  (27.4) | 67  (24.4) | 0.932 | 1 | 0.334 |
| Good health status | 699  (77.8) | 485  (78.5) | 214 (76.4) | 0.505 | 1 | 0.777 | 489  (78.2) | 210 (76.9) | 0.308 | 1 | 0.857 | 488  (78.3) | 211  (76.7) | 0.369 | 1 | 0.832 |
| Poor relationship with friends/family members | 376  (41.9) | 246  (39.8) | 130  (46.4) | 3.472 | 1 | 0.062 | 251  (40.2) | 125 (45.8) | 2.472 | 1 | 0.116 | 250  (40.1) | 126  (45.8) | 2.538 | 1 | 0.111 |
| Irritability personal characteristics | 598  (66.6) | 375  (60.7) | 223  (79.6) | 31.148 | 1 | **<0.001** | 382  (61.1) | 216 (79.1) | 27.675 | 1 | **<0.001** | 380  (61.0) | 218  (79.3) | 28.649 | 1 | **<0.001** |
| Poor family support | 208  (23.2) | 147  (23.8) | 61  (21.8) | 0.433 | 1 | 0.510 | 151  (24.2) | 57  (20.9) | 1.149 | 1 | 0.284 | 150  (24.1) | 58  (21.1) | 0.956 | 1 | 0.328 |
| Stress event | 143  (15.9) | 76  (12.3) | 67  (23.9) | 19.470 | 1 | **<0.001** | 80  (12.8) | 63  (23.1) | 14.989 | 1 | **<0.001** | 80  (12.8) | 63  (22.9) | 14.444 | 1 | **<0.001** |
| Less interpersonal communication | 615  (68.5) | 398  (64.4) | 217 (77.5) | 15.319 | 1 | **<0.001** | 402  (64.3) | 213 (78.0) | 16.528 | 1 | **<0.001** | 401  (64.4) | 214  (77.8) | 15.996 | 1 | **<0.001** |
|  |  |  |  |  |  |  |  |  |  |  |  |  |  |  |  |  |
|  | **Mean (SD)** | **Mean (SD)** | **Mean (SD)** | **t/z** | **df** | **p** | **Mean (SD)** | **Mean (SD)** | **t/z** | **df** | **p** | **Mean (SD)** | **Mean (SD)** | **t/z** | **df** | **p** |
| Age (years) | 37.22  (16.99) | 38.48 (15.95) | 34.44 (18.83) | -4.511 | ---* | **<0.001** | 38.62  (15.92) | 34.00 (18.87) | -5.028 | ---* | **<0.001** | 38.59 (15.90) | 34.12 (18.91) | -4.910 | ---* | **<0.001** |
| Education background (years) | 13.09  (3.66) | 13.35  (3.58) | 12.50 (3.79) | 3.250 | 897 | **0.001** | 13.37  (3.59) | 12.42 (3.75) | 3.624 | 897 | **<0.001** | 13.36  (3.59) | 12.45  (3.76) | 3.459 | 897 | **0.001** |
| Illness duration (years) | 7.55  (8.54) | 8.38  (8.81) | 5.73 (7.60) | -4.779 | ---* | **<0.001** | 8.30  (8.79) | 5.831  (7.66) | -4.362 | ---* | **<0.001** | 8.33  (8.79) | 5.79  (7.65) | -4.566 | ---* | **<0.001** |
| First episode age (years) | 29.88  (15.05) | 30.41  (13.96) | 28.69 (17.19) | -3.726 | ---* | **<0.001** | 30.63 (14.02) | 28.15 (17.09) | -4.488 | ---* | **<0.001** | 30.57  (13.97) | 28.31  (17.19) | -4.316 | ---* | **<0.001** |
| CRP (μg/L) | 0.57  (1.47) | 0.56  (1.52) | 0.59 (1.34) | -1.191 | ---* | 0.234 | 0.56  (1.51) | 0.59 (1.36) | -1.810 | ---* | 0.070 | 0.56  (1.52) | 0.59  (1.35) | -1.670 | ---* | 0.095 |
| Cortisone (nmol/L) | 16.68  (11.41) | 17.16  (12.27) | 15.62  (9.14) | -2.012 | ---* | **0.044** | 17.29  (12.18) | 15.29 (9.27) | -2.781 | ---* | **0.005** | 17.28 (12.20) | 15.31  (9.25) | -2.740 | ---* | **0.006** |
| ACTH (pg/ml) | 31.84  (28.77) | 33.67  (30.15) | 27.81  (25.03) | -2.631 | ---* | **0.009** | 33.73  (30.04) | 27.49 (25.14) | -2.931 | ---* | **0.003** | 33.70  (30.05) | 27.61  (25.17) | -2.853 | ---* | **0.004** |
| Testosterone (nmol/L) | 181.51  (226.47) | 197.35 (237.64) | 146.53 (195.50) | -2.956 | ---* | **0.003** | 198.37  (237.52) | 142.91 (193.83) | -3.343 | ---* | **0.001** | 198.22 (237.86) | 143.65  (193.39) | -3.233 | ---* | **0.001** |
| HAMD-24 total | 13.76  (10.76) | 9.42 (7.91) | 23.32 (10.03) | -17.595 | ---* | **<0.001** | 9.67  (8.26) | 23.10 (9.98) | -17.013 | ---* | **<0.001** | 9.65  (8.26) | 23.05  (9.96) | -17.060 | ---* | **<0.001** |
| HAMA total | 7.86  (8.02) | 5.62 (6.64) | 12.82  (8.59) | -13.371 | ---* | **<0.001** | 5.82  (6.91) | 12.55 (8.44) | -12.722 | ---* | **<0.001** | 5.81  (6.91) | 12.54  (8.41) | -12.820 | ---* | **<0.001** |
| BPRS total | 32.48  (10.22) | 32.84 (10.58) | 31.69 (9.37) | 1.572 | 897 | **<0.001** | 32.96 (10.57) | 31.40 (9.31) | 2.108 | 897 | **0.027** | 32.96  (10.59) | 31.40 (9.28) | 2.118 | 897 | **0.034** |
| YMRS total | 14.36  (11.72) | 17.71  (11.75) | 6.96  (7.52) | -12.698 | ---* | **<0.001** | 17.71  (11.71) | 6.67  (7.31) | -13.063 | ---* | **<0.001** | 17.74  (11.72) | 6.68  (7.31) | -13.110 | ---* | **<0.001** |

Figure 1. Distribution of propensity score of suicidal ideations


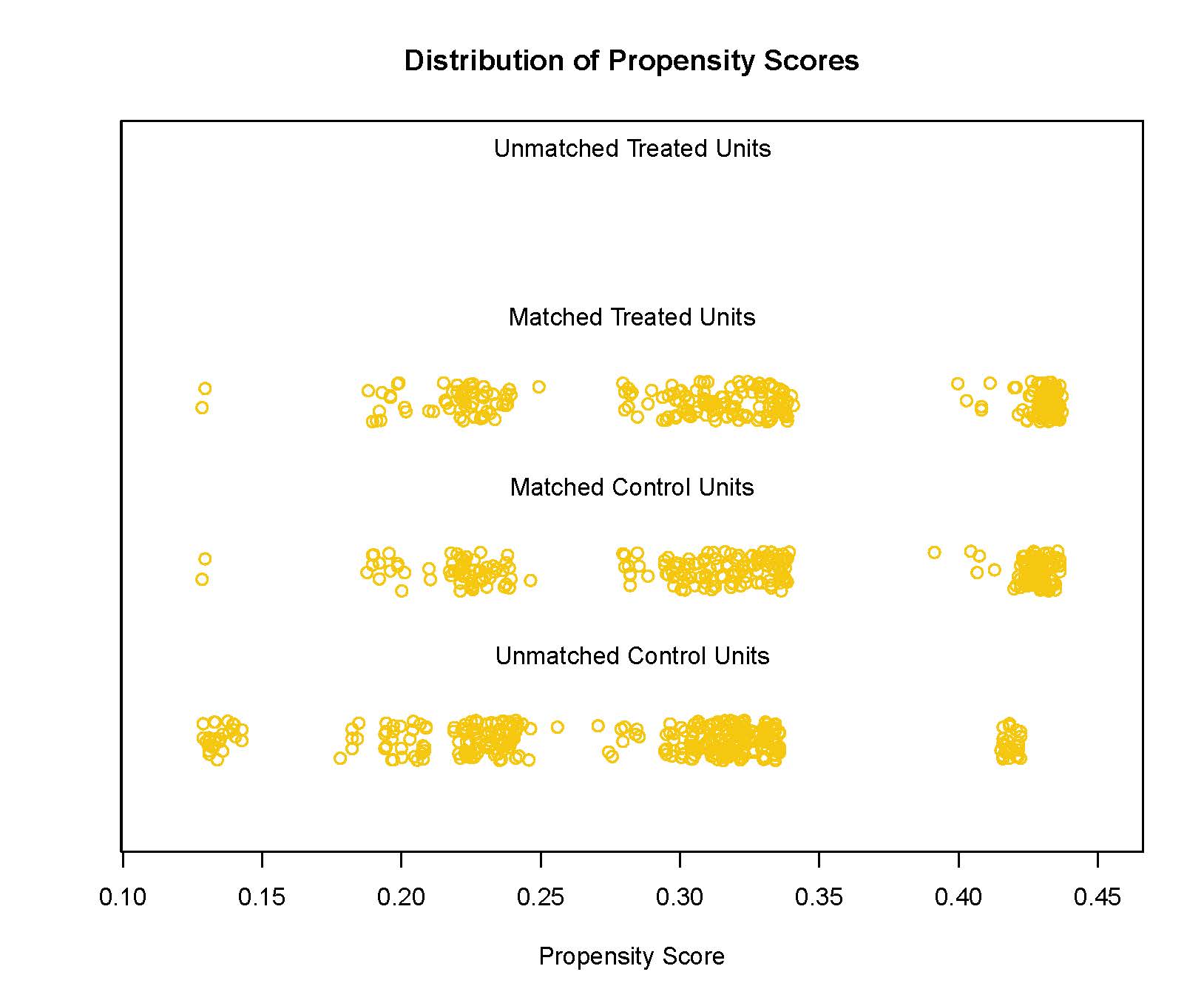


Figure 2. Distribution of propensity score of suicide plan


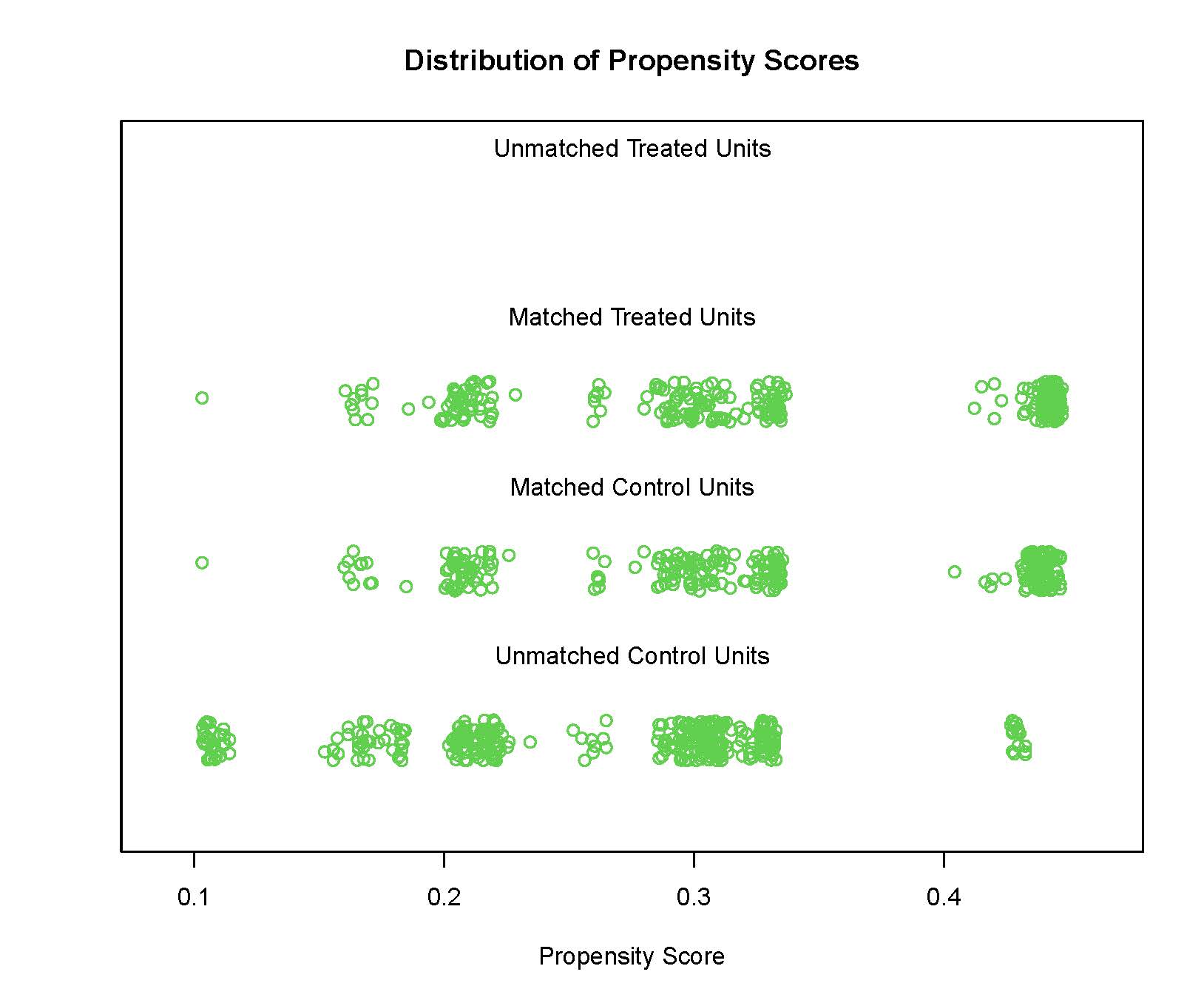


Figure 3. Distribution of propensity score of suicide attempts


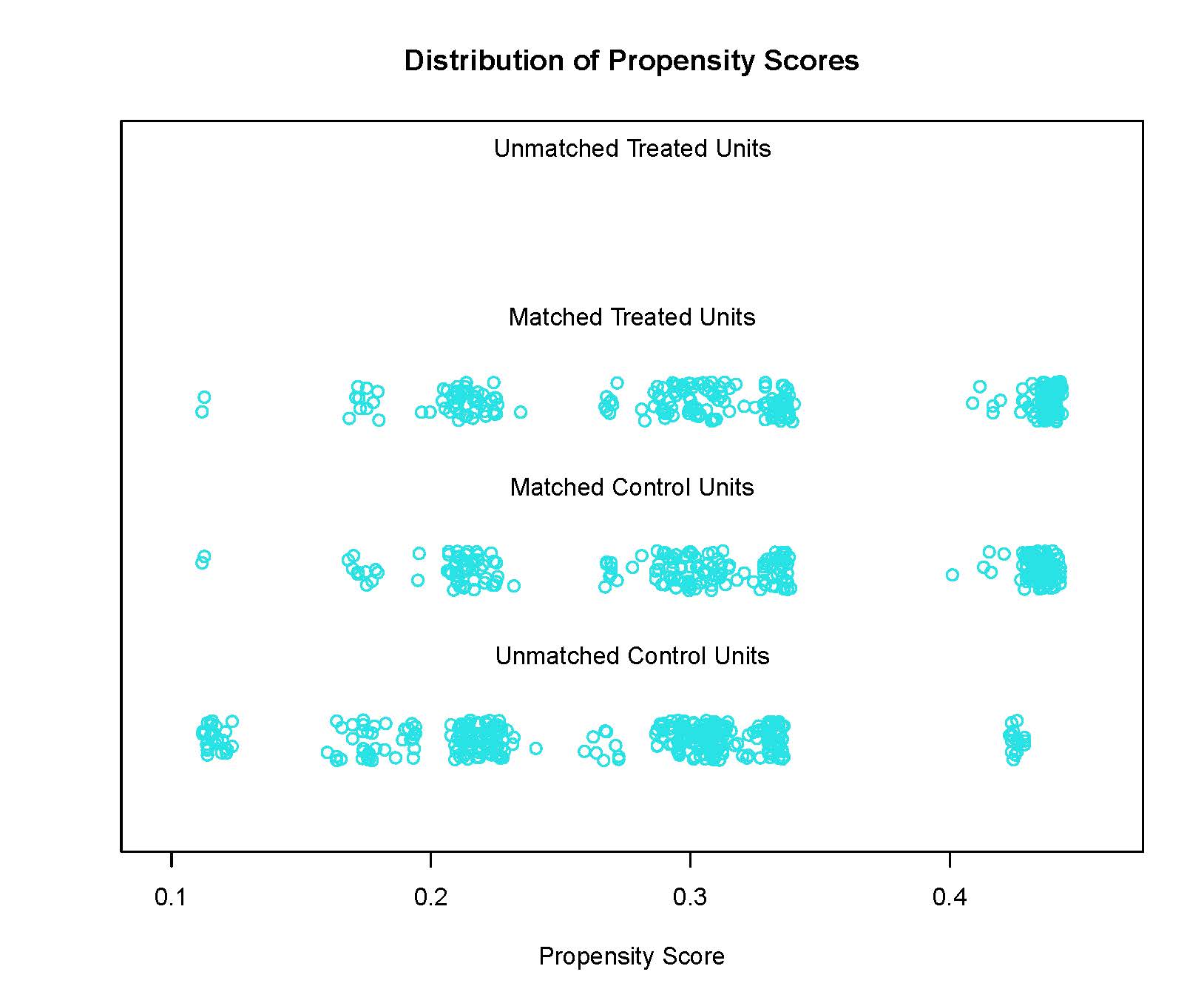

Supplement: Supplementary file 1 — supplementary naterials [file 41398_2023_2675_MOESM1_ESM.docx]
